# Agreement of Blood Spot Card Measurements of Vitamin D Levels with Serum, Whole Blood Specimen Types and a Dietary Recall Instrument

**DOI:** 10.1371/journal.pone.0016602

**Published:** 2011-01-31

**Authors:** Emma K. Larkin, Tebeb Gebretsadik, Nathan Koestner, Mark S. Newman, Zhouwen Liu, Kecia N. Carroll, Patricia Minton, Kim Woodward, Tina V. Hartert

**Affiliations:** 1 Division of Allergy, Pulmonary and Critical Care, Department of Medicine, Vanderbilt University, Nashville, Tennessee, United States of America; 2 Department of Biostatistics, Vanderbilt University, Nashville, Tennessee, United States of America; 3 ZRT Laboratory, Beaverton, Oregon, United States of America; 4 Department of Pediatrics, Vanderbilt University, Nashville, Tennessee, United States of America; Indiana University, United States of America

## Abstract

**Background:**

The ability to measure 25-hydroxyvitamin D (25OHD) levels from blood spot cards can simplify sample collection versus samples obtained by venipuncture, particularly in populations in whom it is difficult to draw blood. We sought to validate the use of blood spot samples for the measurement of 25OHD compared to serum or whole blood samples and correlate the measured levels with intake estimated from dietary recall.

**Methods:**

Utilizing 109 biological mothers of infants enrolled in the Tennessee Children's Respiratory Initiative cohort, we measured 25OHD levels through highly selective liquid chromatography–tandem mass spectrometry on samples from blood spot cards, serum, and whole blood collected at enrollment. Dietary questionnaires (n = 65) were used to assess 25OHD intake by dietary recall. Sample collection measures were assessed for agreement and 25OHD levels for association with dietary 25OHD intake.

**Results:**

The mean absolute differences (95%CI) in 25OHD levels measured between whole blood and blood spot (n = 50 pairs) or serum and blood spot (n = 20) were 3.2 (95%CI:1.6, 4.8) ng/ml and 1.5 (95%CI:−0.5,3.4) ng/mL. Intake by dietary recall was marginally associated with 25OHD levels after adjustment for current smoking and race in linear regression.

**Discussion:**

25OHD levels determined by mass spectrometry from blood spot cards, serum and whole blood show relatively good agreement, although 25OHD levels are slightly lower when measured by blood spot cards. Blood spot samples are a less invasive means of obtaining 25OHD measurements, particularly in large population-based samples, or among children when venipuncture may decrease study participation.

## Introduction

Vitamin D is becoming recognized for the biological role played in development of a wide variety of conditions including bone fractures[1], resistance to microbial infections[2], cardiovascular disease [3], [4], cancer[5] and asthma[6], [7]. Vitamin D can come from dietary sourcesincluding fortified foods and supplements. More abundantly, vitamin D is synthesized from 7-dehydrocholesterol by ultraviolet B exposure to the skin and converted to a measurable circulating metabolite 25-hydroxy vitamin D (25OHD) in the liver. Total 25OHD has 25-hydroxy vitamin D_2_ and 25-hydroxy D_3_ as its constituent parts.

There has been longstanding debate in the literature about how to accurately measure 25OHD [8]–[10]. The lipophilic hydrophobic properties of vitamin D, coupled with its tight affinity to vitamin D binding protein, have made it challenging to measure. Early techniques to detect 25OHD include competitive binding protein assays [11], [12] which are susceptible to matrix effects and cross-reactivity with other Vitamin D metabolites and can bias measurement of 25OHD [8]. Other enzyme immunoassays and chemiluminescence based tests [13], [14] have been automated for large scale processing to handle the increased demand of clinical testing, which were not possible with the gas chromatographic or high performance liquid chromatography methods, both of which have been considered the best tools for 25OHD measurement [15]–[18]. More recently, highly selective liquid chromatography–tandem mass spectrometry (LC-MS/MS) is emerging as a respected assay due to its high specificity in teasing apart 25OHD_2_ and 25OHD_3_.[15], [19]

Typically, 25OHD assays utilize plasma or serum which may be difficult to collect in large epidemiological studies or among populations in whom blood draws decrease study participation, such as in infants and children. For this reason, we sought to evaluate the agreement in measurement of vitamin D by LC-MS/MS from a blood spot card, prepared in the lab with vitamin D measurements by LC-MS/MS from serum or whole blood samples. We also wanted to assess whether a food frequency questionnaire that estimated dietary vitamin D intake provided an adequate proxy measure for quantified levels of circulating Vitamin D.

## Methods

### Study population

The Tennessee Children's Respiratory Initiative is a longitudinal study of mother-infant dyads designed to understand the relationship between infant respiratory infections and asthma and atopic diseases. Term, non-low birth weight previously healthy infants and their biological mothers were enrolled during infancy during 2004–2008, when the infant was treated for viral lower or upper respiratory tract infection (N = 630), as has been previously described.[20] A subset of 109 mothers with available blood specimens had their 25-hydroxyvitamin D levels determined through the use of laboratory created blood spot cards. Forty-seven percent (50/109) of these women had their 25OHD levels additionally assayed through the use of whole blood and 19% (20/109) had levels assayed using an available serum sample. Maternal samples were collected only during the fall, winter and spring seasons, reflecting the study enrollment periods during viral seasons.

This research was approved by the Institutional Review Board at Vanderbilt University. Written informed consent was obtained from participants within this study.

### Blood Spot preparation

In the laboratory, using whole blood specimens stored at −80 degrees from mothers in the study, between 3 and 12 non-overlapping drops of the whole blood were pipetted onto laboratory provided blood spot cards with a pre-stamped circle. Blood spots were air dried for at least 30 minutes before closing the flap on the blood spot card. Fully dried samples were refrigerated and sent in batches to ZRT Laboratory, (Beaverton, OR) for vitamin D assay.

### Measurement of 25OHD

25-OHD was extracted from 6mm spots and derivatized for analysis by LC-MS/MS using previously described methods [21], [22] which were modified to allow for automation. Spots were punched from dried blood spot cards (Wallac MultiPuncher) and reconstituted with 600 ul of deionized water. 600 ul of methanol containing internal standard (D4-25-hydroxyvitamin D3) was then added to precipitate proteins and the samples were vortexed. 900 ul of the supernatant was extracted with C18 solid phase extraction. Extracted samples were derivatized with 200 ul of 0.1 mg/ml PTAD (4-phenyl-1,2,4-triazoline-3,5-dione) at room temperature for 10 minutes. Derivatized samples were blown to dryness with nitrogen and reconstituted with 50 ul of methanol and 20 ul injected into the LC-MS/MS system (Varian). The vitamin D assay was repeated by ZRT labs using the above procedure with 20 uL of whole blood or 10 ul serum for comparison. Separate calibrations were run for each sample type.

### Vitamin D Dietary Intake Ascertainment

The Block 2000 Brief Food Questionnaire (Block Dietary Data Systems, Berkeley, California) was available on 65 women who also had available blood spot samples from the enrollment visit. This 70 item test asks about usual eating habits in the past year as well as about multivitamin supplementation, from which vitamin D dietary intake is calculated. It is based on an earlier validated brief food frequency questionnaire[23]. A composite variable representing total vitamin D intake was created by summing the food and supplement amounts of vitamin D.

### Variable Definition

25OHD levels were analyzed as continuous measures and subjects were also grouped in categories defined as: sufficient ≥30 ng/mL, insufficient, ≥20–29 ng/mL and deficient <20 ng/mL. These categories were selected from Holick 2007 who synthesized existing expert opinion with biologic evidence from observed relationships between parathyroid hormone and vitamin D and calcium transport and Vitamin D [24]. Ethnicity and education were dichotomized into European-American and non European-American groups and high school graduate or less compared to college attendance. Maternal season of study for time of vitamin D ascertainment was categorized as fall (September through November), winter (December through February) or spring (March through May) with no recruitment during the summer non-viral seasons.

### Statistical Methods

We compared the agreements of results between vitamin D measurements by LC-MS/MS utilizing blood spot samples, whole blood samples, or serum samples in two ways. First, we used Bland Altman plots with vitamin D means plotted on the horizontal axis and the differences plotted on the vertical axis for visual inspection of the amount of disagreement between the two measures. Horizontal lines are drawn at the mean difference, and at the limits of agreement, which are defined as the mean difference plus and minus 1.96 times the standard deviation of the differences. Next, we calculated the absolute difference and the percent difference between the sample types to estimate systematic bias. We used paired t-test difference analysis with log transformation of 25OHD levels. We also assessed correlations between the different measures using Spearman rank correlation coefficient; all pairwise Spearman correlation coefficients were ≥0.9, p<0.001. We used the binomial Wilson approximation for calculation of 95% confidence interval of proportions by category definitions [25]. Multivariable linear regression was used to assess the association between vitamin D intake and 25OHD levels by blood spot after adjusting for *a priori* chosen covariates including maternal age, maternal race and current smoking. The association of serum 25OH by blood spot with Vitamin D intake was analyzed using separate models from food sources, from supplements and the sum. Maternal 25OHD levels were log transformed for normality. In addition we performed Box-Cox power transformation as a sensitivity analysis. As results were similar, we present log transformed analysis for ease of beta coefficient interpretation.

## Results

The characteristics of the study population are described in Table 1. Of the 109 women with 25OHD levels measured utilizing whole blood spot cards, there were 50 women with both blood spot and whole blood samples and 20 women with serum and whole blood samples, or serum and blood spot samples. For all 109 samples, the mean maternal age was 26.5±6.6 SD years. Approximately 80% were European-American and approximately 30% were smokers. Sixty percent of maternal measurements were obtained during winter months. Population characteristics did not vary by the subset of available data.

**Table 1:** Subject Characteristics of Mothers with Vitamin D Measurements Determined by LCMS/MS Used in Pairwise Comparisons.

Characteristic		N (%) or Mean (SD)
Number of mothers with blood spot cards and whole blood		N = 50
Number of mothers with blood spot cards and serum		N = 20
Number of mothers with blood spot cards and food frequency questionnaires		N = 65
Mean maternal age at measurement		27.4 (5.6)
Race		
	European-American	90 (83%)
	Non-European American	17 (17%)
Season sample obtained		
	Winter	69 (63%)
	Fall/Spring	40 (37%)
Current maternal smoking		30 (28%)
Education		
	≥12 years of education	83 (83%)
	<12 years	17 (17%)
Mean weeks postpartum		14 (12)
Mean 25OHD levels, ng/mL		25 (12)
Vitamin D status based on blood spot (N = 109)		
	Sufficient ≥30 ng/mL	30 (28%)
	Insufficient: 20–30 ng/mL	37 (34%)
	Deficient <20 ng/mL	42 (39%)
Mean dietary intake, IU		151 (114)
Supplemental use (N = 65)		
	None	33 (51%)
	1–399 IU	9 (16%)
	≥400 IU	23 (36%)
Supplemental + diet mean intake, IU		331 (238)

Total vitamin D levels, measured by blood spot cards, averaged 25±12 ng/mL. All of the 25OHD_2_ levels were <4 ng/mL and, thus, the total 25OHD values consist almost entirely of 25OHD_3_ levels. Sixty-five mothers had available paired food frequency questionnaire (FFQ) data and available blood spot vitamin D measures. The average dietary intake of vitamin D from food sources was 151.0±114.2 IU. The median supplemental intake was 0 with an interquartile range [IQR] of [0–400 IU]. The composite measure of total vitamin D intake was 331±238 IU.

**Figure 1:**
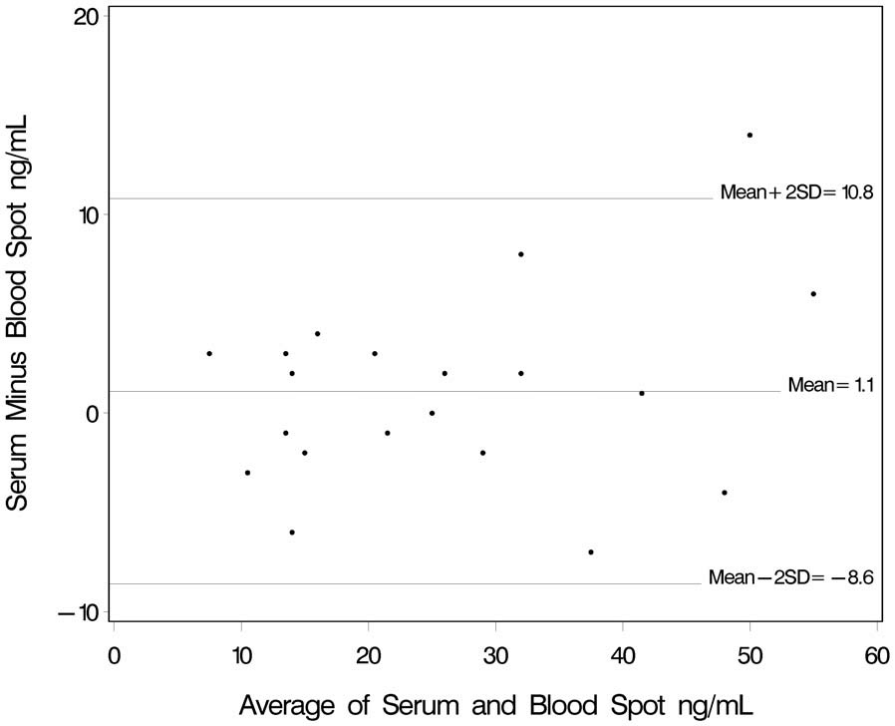
Bland-Altman plots of 25OHD measurements comparing blood spot cards to serum, using liquid chromatography–tandem mass spectrometry.

**Figure 2:**
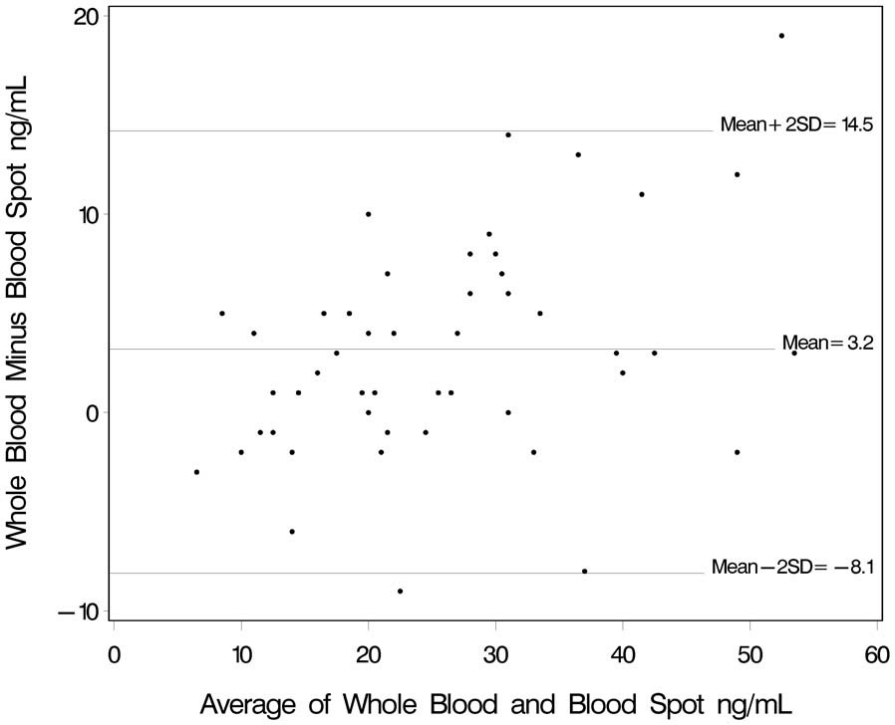
Bland-Altman plots of 25OHD measurements comparing blood spot cards to whole blood, using liquid chromatography–tandem mass spectrometry.

Agreement in 25OHD measurements using blood spot, serum and whole blood samples were evaluated using Bland-Altman plots, and are presented in figures 1 and 2. In the serum versus blood spot comparison of figure 1, only one point falls out the two standard deviation interval. Blood spot measurements were on average 1.1 ng/mL lower with a 3.3 percent difference that was not statistically significant (95% CI: −6.3–12.1%; p = .48).

Similarly in figure 2, only 2 observations fell outside of the two standard deviation bounds for blood spot and whole blood measurements. The blood spot 25OHD measurements were on average 3.2 ng/mL lower than whole blood, which corresponds to an 11% difference (95% CI: 3.8%–18.3%; p = 0.003).

Sixty five mothers had both food frequency questionnaire data on vitamin D dietary intake and biospecimens available for measurement of vitamin D levels utilizing blood spot cards. Dietary intake of vitamin D by 24 hour recall was not associated with vitamin D levels in either univariate analyses or multivariable regression, after adjusting for maternal age, smoking, and race. Table 2 shows the parameter estimates for models that consider dietary intake from food, dietary intake from supplements, and dietary intake from the sum of food and supplemental intake in separate regression models. Log transformed vitamin D intake was marginally associated with a 0.24 ng/mL increase in 25OHD levels (p = 0.05). However when we applied Box Cox transformation for normality of residuals of multivariable linear regression, the statistical significance was attenuated (p = 0.08).

**Table 2:** Relationship between Vitamin D intake and supplemental vitamin D use on circulating vitamin D levels.

Intake Measurement	Coefficient (95% CI)*	P value**
Vitamin D intake	0.11 (−0.03,0.25)	0.14
(IQR difference: 134 units)		
Supplemental vitamin D	0.19 (−0.07,0.46)	0.16
(IQR difference: 400 units)		
Sum of Dietary and Supplement	0.24 (0.0048, 0.48)	0.05
(IQR difference: 439 units)		

*Multivariable linear regression was used to calculate Vitamin D intake coefficient and 95%CI. Maternal vitamin D (blood spot card) was natural log transformed, adjusting for race/ethnicity, smoking status and maternal age.

**all p value remained >0.05 after Box Cox transformation for normality of residuals.

## Discussion

This study examines the agreement between vitamin D measurements by LC-MS/MS comparing dried blood spot, serum and whole blood samples. Measurement of vitamin D using the three specimen types showed good, though imperfect, agreement. The measurement of vitamin D using blood spot card samples is a reliable means of collecting and measuring vitamin D levels. Blood spot measurements were on average lower than both whole blood and serum measurements. In the absence of a gold standard, we can assess extent of differences, but we do not have a known true measure to identify whether the bias is with blood spot for example. However, the results are consistent at both higher and lower levels, thus the relative vitamin D measurements are consistent across a spectrum of values.

The highest difference observed (11%) was between blood spot and whole blood levels, where the whole blood levels may not have adequately been corrected for the whole blood hematocrit. Alternatively, it is possible that some degradation of vitamin D may have occurred in the drying of the blood spot card as suggested by Eyles et al (2010), who demonstrated excellent correlation between cord blood 25OHD levels compared to dried blood spot cards [26]. Because whole blood was available on a larger number of samples, it provided additional information in evaluating dried blood spots. We note that the most relevant comparison is between serum and blood spot samples because both these assays are commercially available and run routinely.

For purposes of epidemiological studies, rather than clinical diagnoses, an average difference of 1.1 ng/mL based on the difference between serum and blood spot cards may not substantially affect the prevalence of insufficiency and deficiency. The point estimates and 95% confidence intervals for the prevalences of sufficiency (28% 95%CI: 20–37%), insufficiency (34% 95%CI: 26–43%) and deficiency (39% 95% CI: 30–48%) overlap with point estimates when a constant of 1.1 is added to address the lower values of blood spots relative to serum levels (32%, 34%, 34% respectively). Furthermore, a categorization approach assumes that the threshold values are intractable, when commentary has begun to suggest that the reference ranges for optimal benefit may be too low [27] and depend on the disease or population characteristics being studied. Based on current studies, theInstitute of Medicine even suggested the opposite, that levels greater than or equal to 20 ng/mL are sufficient for bone health where causality has been strongly established [28]. It has even been proposed that thresholds be determined by assay type that accounts for measurement variability [29]. The findings from this study would support such an approach.

Many prior analyses have explored the relationship between vitamin D measurements conducted using different assay types [9], [16], [30]–[32]. For example, a comparison of 25OHD levels from serum measured on HPLC, LC-MS/MS, and RIA, showed inter-assay differences of 2.9 to 5.1 ng/mL, where LC-MS/MS and RIA methods were higher than HPLC[32]. A calibration equation that corrected for the systematic bias did not entirely eliminate the inter-laboratory differences. A similar upward bias was seen comparing LC-MS/MS to seven commercially available assays and also concluded that calibration did not eliminate interassay differences [33]. The National Institute of Standards and Technology has developed a standard measurement (certified in July 2009) that will help improve the measurement of vitamin D by encouraging inter-laboratory consistency agreement. This standard was not available at the time the samples were run.

Because maternal dietary vitamin D intake was also available, we determined the agreement in dietary intake as determined by food frequency questionnaires with measured vitamin D levels. In this population, the major source of vitamin D was D3, converted by the skin from sunlight exposure. While measurements were broken down by the 25OHD_2_ and 25OHD_3_ components, all the vitamin 25OHD_2_ measurements were below the minimum detectable level. Thus, dietary intake information using FFQs in this population did not correlate with measured vitamin D levels, likely because the major source of vitamin D in this population is sunlight and not food sources. Such a finding is consistent with other studies that have found a low correlation between dietary intake and serum levels[34], [35].

In this study, we used a single sample to measure vitamin D levels. Several studies have demonstrated that season of measurement explains the greatest variability in vitamin D measurements. However, in studies that have compared vitamin D measurements at several seasonal time points, the correlation between two measures was high 0.55–0.80 and was greatest when measured 1 year apart during the same season [36]. Measurements taken over 14 years also demonstrated correlations of .39–0.52, with most individuals at the extreme quintiles remaining in the same quintile over time[36].

This study has several limitations. Blood spot cards were prepared in the laboratory and not tested in the field using a finger or heel prick, and this could change the results. In this analysis, 25OHD_2_ levels were less than the detectable limit (4 ng/mL), and, thus, results driven by the 25OHD_3_. The lack of detection of 25OHD_2_ in our study is likely to be due to the lack of high supplementation in the population, which was noted on the food frequency questionnaire, because of this, however, assessment of the agreement of 25OHD_2_ can therefore not be made in this study. In a study conducted by ZRT Laboratory, which looked at 25OHD_2_ and 25OHD_3_ levels in blood spot cards, the authors find evidence suggesting that there is proportional bias with 25OHD_2_ blood spot cards compared to serum [22].

Blood spot cards enable an easy, safe, and practical way to screen for vitamin D deficiencies in at-risk populations where venipuncture is impractical including, elderly and pediatric populations, rural clinics, and developing countries. The assay variability due to the type of specimen analyzed was comparable to variability observed when comparing across laboratory methods. The introduction of the standard will certainly assist in addressing laboratory differences, calibrating vitamin D measurements to better measure true underlying levels. Blood spot cards also brings us one significant step closer to mass screening for vitamin D deficiency, something that has so far not been practical despite the great importance of vitamin D to the health of the skeletal, immune, and cardiovascular systems in all phases of the human life cycle.
